# Contact Guidance Effect and Prevention of Microfouling on a Beta Titanium Alloy Surface Structured by Electron-Beam Technology

**DOI:** 10.3390/nano11061474

**Published:** 2021-06-02

**Authors:** Sara Ferraris, Fernando Warchomicka, Jacopo Barberi, Andrea Cochis, Alessandro Calogero Scalia, Silvia Spriano

**Affiliations:** 1 Department of Applied Science and Technology, Politecnico di Torino, 10129 Torino, Italy; jacopo.barberi@polito.it (J.B.); silvia.spriano@polito.it (S.S.); 2 Institute of Materials Science, Joining and Forming, Graz University of Technology, A-8010 GRAZ, Austria; fernando.warchomicka@tugraz.at; 3 Department of Health Sciences, Università del Piemonte Orientale, 28100 Novara, Italy; andrea.cochis@med.uniupo.it (A.C.); alessandro.scalia@uniupo.it (A.C.S.); 4 Center for Translational Research on Autoimmune & Allergic Diseases—CAAD, 28100 Novara, Italy

**Keywords:** beta titanium alloys, Ti15Mo, surface modification, electron beam technique, nano-topography, antifouling, biofilm

## Abstract

Nano- and micro-structuring of implantable materials constitute a promising approach to introduce mechanical contact guidance effect, drive cells colonization, as well as to prevent bacteria adhesion and biofilm aggregation, through antifouling topography. Accordingly, this paper aims to extend the application of e-beam surface texturing and nano-structuring to the beta titanium alloys, which are of great interest for biomedical implants because of the low Young modulus and the reduction of the stress shielding effect. The paper shows that surface texturing on the micro-scale (micro-grooves) is functional to a contact guidance effect on gingival fibroblasts. Moreover, nano-structuring, derived from the e-beam surface treatment, is effective to prevent microfouling. In fact, human fibroblasts were cultivated directly onto grooved specimens showing to sense the surface micro-structure thus spreading following the grooves’ orientation. Moreover, *Staphylococcus aureus* colonies adhesion was prevented by the nano-topographies in comparison to the mirror-polished control, thus demonstrating promising antifouling properties. Furthermore, the research goes into detail to understand the mechanism of microfouling prevention due to nano-topography and microstructure.

## 1. Introduction

Titanium and its alloys are the most widespread materials used for load bearing and bone contact in biomedical implants, due to their good mechanical properties and biocompatibility. Among them, commercially pure titanium (Ti-cp) and Ti6Al4V alloys are the most diffused. However, some criticisms are still present. One is related to the elastic modulus, which is lower than that of stainless steel and Co-Cr alloys, but significantly higher than the one of bone. The second criticism is related to the potential release of toxic elements (e.g., Al and V [1]). In this context, Ti-Mo alloys are gaining interest due to their lower elastic modulus and the absence of potentially toxic alloying elements [2,3,4]. Moreover, these alloys present an increased corrosion resistance evidenced also in the oral environment [5,6]. That is why the focus of this work is on beta Ti-Mo alloys.

In addition to biocompatibility and mechanical compatibility, the ability to counteract bacterial adhesion is one of the main features desired for biomedical implants. In fact, bacterial infections are one of the main causes of implant failures in both the orthopedic and dental fields [7,8]. Since the majority of implant-related infections are associated with biofilm formation on the implant surface, several strategies have been investigated to reduce bacterial adhesion on titanium surfaces. Among them, the localized release of antibiotics, organic or inorganic antibacterial agents, such as metal ions or nanoparticles, can be cited [9,10,11,12]. Finally, topographical modifications should be synergistically designed to be suitable for both the optimization of tissue integration and infection passive prevention, such as through surface nanotextures [13,14,15,16,17,18,19,20,21,22,23].

In the case of transmucosal and percutaneous dental or orthopedic implants, an effective soft tissue sealing around the implant is crucial for good functionality and prevention of bacterial infiltration [24], in addition to bone integration. Even if surface modifications for soft tissue contact are less explored than the ones for bone integration, some research works investigate the possibility of topographical, chemical or biological modifications able to guide soft tissue onto titanium surfaces [25]. Some of them have been already applied to commercial dental implants. Oriented micro-grooves can effectively stimulate fibroblast adhesion and orientation (contact guidance phenomenon) [26,27] for an effective gum sealing, limited epithelial downgrowth and limited bacterial infiltration, as demonstrated in dental implants [28]. In this context, the authors have already explored the ability of submicrometric grooves and keratin nanofibers to effectively guide gingival fibroblasts onto commercially pure titanium surfaces, limiting also bacterial adhesion [29,30,31]. On alpha titanium (Ti-cp) and an alpha-beta titanium alloy (Ti6Al4V), the authors investigated the use of electron beam technique to design a specific surface to control the contact guidance effect and prevent microfouling. The former effect is due to surface texturing (micro-grooves) and the latter to surface nano-structuring (high density of grain boundaries and connected topography) due to the e-beam process [32,33].

In this paper, it is demonstrated that an electron beam surface treatment on a beta alloy can effectively promote both the contact guidance effect and microfouling prevention. The mechanism of anti-fouling of bacteria due to surface nano-structuring is investigated much more in detail. Ti-Mo alloys were selected as beta alloys, because even if they are still less studied than Ti-cp and Ti6Al4V, they are promising as possible bone implants [34,35] and specific surface modifications are needed to improve their surface properties [36]. On the other hand, almost no specific research is reported in the literature for increasing the soft tissue adhesion of these alloys, to the best of the authors’ knowledge.

## 2. Materials and Methods

### 2.1. Samples Preparation

A metastable beta-titanium alloy Ti15Mo was used in plate shape (10 mm × 15 mm × 2 mm), with a chemical composition in wt.% of 15.8% Mo, 0.05% Fe and 0.15% rest Ti [37]. Samples were mirror polished with final step of 1 µm diamond (Struers Tegramin-30, Struers, Copenhagen, Denmark) and washed in an ultrasonic bath with acetone (5 min) and water (10 min, twice).

Surface structuring was performed with an Electron Beam Welding machine (EBG 45-150 k14, Probeam GmbH & Co. KGaA, Gilching, Germany), performing the same route of processing reported by the authors for commercially pure titanium and Ti6Al4V alloys [32,33]. In this work, grooves of 10 and 30 µm were obtained on Ti15Mo substrates, using as main process parameters an accelerated voltage of 150 kV, a beam current of 0.8 mA and a beam travel speed of 853 and 3333 mm/s for grooves of 30 and 10 µm, respectively. All the experiments were carried out in vacuum.

Part of the samples were thermally treated in a dilatometer (Bähr-Thermoanalyse DIL805A/D, Bähr Thermoanalyse GmbH, Hüllhorst, Germany) in a high vacuum atmosphere (<10–5 mbar) to avoid the formation of an oxide layer at the structured area. Samples were heated up to 850 °C at 300 K/min, with a subsequent dwell time of 5 min and then cooled with a constant rate of 20 K/min down to room temperature. The slow cooling from the β-field promotes the formation of α phase. This heat treatment was performed to evaluate the influence of a slow cooling rate on the formation of α in the β matrix, with respect to the expected metastable β phase provoked by fast cooling condition of the process [32,33].

After structuring, the samples were washed in acetone and water in an ultrasonic bath as previously described.

### 2.2. Physico-Chemical Characterizations

Surface topography was investigated by means of Field Emission Scanning Electron Microscopy (FESEM, TESCAN Mira 3, Tescan, Brno, Czech Republic), confocal microscopy (LSM 900, ZEISS, Oberkochen, Germany) and Atomic Force Microscopy (AFM, Innova, Bruker, Billerica, MA, United States). Confocal images were obtained by using 20× and 50× objectives, resulting in 256 × 256 µm and 128 × 128 µm images, respectively. Height maps were obtained from the z-stacks using the ConfoMap software and surface roughness values were obtained according to the ISO 25178. AFM images were collected in tapping mode using Si tips, and scans of 50 × 50 µm, 10 × 10 µm and 3 × 3 µm were performed, resulting in lower and higher magnifications. The data were analyzed using Gwyddion’s free software.

X-Ray Diffraction (XRD, PANalytical X’Pert Pro PW 3040160 Philips, Malvern Panalytical, Egham, UK) equipped with a copper radiation source was used for the investigation of crystalline structure. The XPERT High Score software (2.2b) was used for the analyses of the obtained spectra. Spectra in the 10–120° 2θ range were acquired.

The sessile drop method was employed for determination of the static contact angle of a water drop and consequent evaluation of surface water wettability. A drop (5 µL) of ultrapure water was deposited on the sample surface with a micropipette and the contact angle measured with the instrument software (DSA-100, KRÜSS GmbH, Hamburg, Germany). At least 3 measurements per sample type were performed.

Surface roughness was measured by contact profiler (Intra Touch, Taylor Hobson, Leicester, UK). For each sample, at least 3 measurements, with 5 mm scan length, were performed. Data were elaborated with TaylyMap software and roughness parameters determined according to the ISO 4287 standard.

### 2.3. Biological Characterizations

Prior to performing biological tests, the samples were heat-sterilized for 2 h at 180 °C in the oven and then stored at room temperature, avoiding any surface damage affecting the micro- and nano-structure.

#### 2.3.1. Cell Adhesion and Orientation

Since the final application intended for the developed surfaces is the collar of transmucosal dental implants, human gingival fibroblasts (HGF, ATCC PCS-201-018, American Type Culture Collection, Manassas, VA, USA) were used for the assessment of cell adhesion and orientation on Ti15Mo substrates, as previously reported by the authors for Ti-cp and Ti6Al4V substrates [32,33]. Cells were cultivated at 37 °C, in a 5% CO_2_ atmosphere with alpha-modified Minimal Essential Medium (α-MEM, from Sigma, Milan, Italy) supplemented with 10% fetal bovine serum and 1% antibiotics (penicillin/streptomycin). Once cells reached 80–90% confluence, they were detached (trypsin-EDTA solution, Sigma, Milan, Italy), counted (Bürker chamber), seeded directly onto the specimen’s surface (final density of 5 × 10^3^ cells/specimen) and cultivated for 48 h at 37 °C, 5% CO_2_. The 48 h timepoint was selected on the basis of previous works in order to investigate cells’ adhesion and alignment in a short time. Then, cell shape and orientation were investigated by means of DAPI, phalloidin and vimentin staining to visualize nuclei and cytoskeleton (F-actin and intermediate filaments). Briefly, cells were fixed with 4% paraformaldehyde (in PBS) for 5 min at room temperature, permeabilized 10 min with 0.1% Triton (in PBS) and stained overnight with anti-Vimentin antibody (1:200 in PBS, Abcam UK). Afterwards, samples were marked for 45 min with phalloidin (1:500 in PBS, from AbCam, UK) to visualize F-actin filaments. Finally, cells were washed with PBS and co-stained with 4,6-diamidino-2-phenylindole (DAPI, from Merck, Darmstadt, Germany) to visualize nuclei. Images were collected by fluorescent microscope (Leica DM 6500, Leica Systems, Basel, Switzerland).

#### 2.3.2. Bacterial Adhesion

*Staphylococcus aureus* (SA, ATCC 43300, American Type Culture Collection, Manassas, VA, USA) was used for the bacterial test because it is a strong biofilm former, methicillin- and oxacillin-resistant and frequently involved in bacteria clusters collected from peri-implantitis clinical cases [38]. Bacteria were cultivated at 37 °C overnight in Trypticase Soybean Broth (TSB, Sigma, Milan, Italy); then, bacteria suspension was diluted 1:10 and incubated again at 37 °C for 3 h to achieve the logarithmic growth phase. Lastly, bacteria were diluted in fresh medium in order to reach a final concentration of 1 × 10^5^ cells per ml corresponding to an optical density = 0.001 at 600 nm wavelength [39]. Each test sample was submerged with 1 mL of bacterial suspension and incubated for 90 min at 37 °C at 120 rpm to force the separation between adherent biofilm bacteria and floating planktonic ones [40]. Afterwards, the broth containing planktonic bacteria was removed and replaced with fresh LB medium to cultivate adhered biofilm up to 72 h [40,41]. At each time point (24, 48 and 72 h), the metabolism of adhered biofilm bacteria was evaluated by the metabolic colorimetric alamar blue assay (alamarBlue^®^, Life Technologies, Milan, Italy) [42,43]. Results were expressed as relative fluorescence units (RFU) and alamar solution was used as a blank. Smooth mirror-polished specimens were used as a control and compared with nano-structured test samples.

#### 2.3.3. Statistical Analysis of Data

Experiments were performed in triplicate. Results were statistically analyzed using the SPSS software (v.20.0, IBM, Segrate (MI), Italy). First, data normal distribution and homogeneity of variance were confirmed by the Shapiro–Wilk and Levene tests, respectively; then, groups were compared by the one-way ANOVA using the Tukey’s test as post-hoc analysis. Significant differences were established at *p* < 0.05.

## 3. Results

### 3.1. Surface Topography Investigation

**Figure 1:**
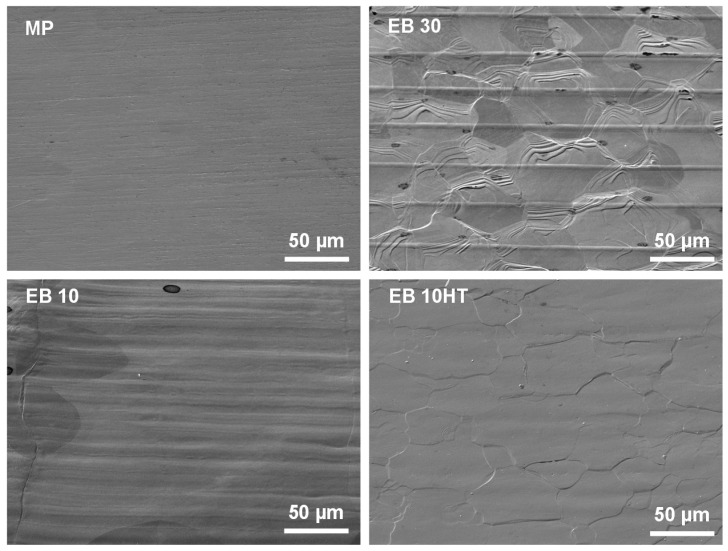
FESEM observations of the studied surfaces: mechanically polished (MP), e-beam structured with grooves 30 or 10 μm large (EB30, EB10) and EB10 thermally treated.

**Figure 2:**
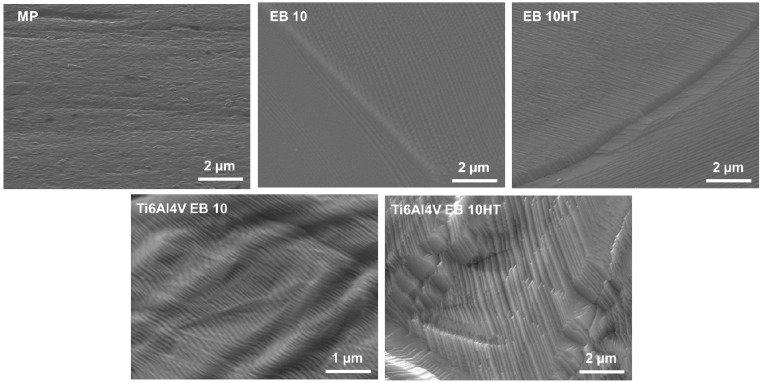
Detail of the nano topography at the surface of Ti15Mo (above) and Ti6Al4V (below) described in previous work [33].

FESEM images, at different magnification, of the pristine and e-beam structured Ti15Mo surfaces are reported in Figure 1 and Figure 2. They were obtained using a pre-tilt of the specimen between 40° and 60° to amplify the topography observations.

As previously reported by the authors for Ti-cp and Ti6Al4V, parallel grooves 10 and 30 µm large are here successfully obtained through e-beam structuring on a beta alloy. Beta grains and grain boundaries are well visible on both the EB10 and EB30 surfaces. The structured area shows smaller grains than the unstructured area due to the quick heating and solidification of the area subjected to the electron beam. After surface structuring, a waved area can be observed at the grain boundaries of the grains, related to the growing of the grain during solidification. The surface of the structured area additionally shows a typical nano-topography composed of steps or terraces, as observed in Figure 2. The heat treatment, performed after e-beam surface structuring, makes the grains and grain boundaries much more visible (Figure 1) and changes the morphology of the nano-topography and defects at the surface, showing a more pronounced plate-like shape. A similar effect was observed on Ti6Al4V, as illustrated in Figure 2 and described in previous work [33]. This surface morphology has been barely studied in metals; some recent works on oxide formation in tungsten [44,45] and thin films [46] aim to manipulate the properties of the material by a similar terrace/stair formation.

**Figure 3:**
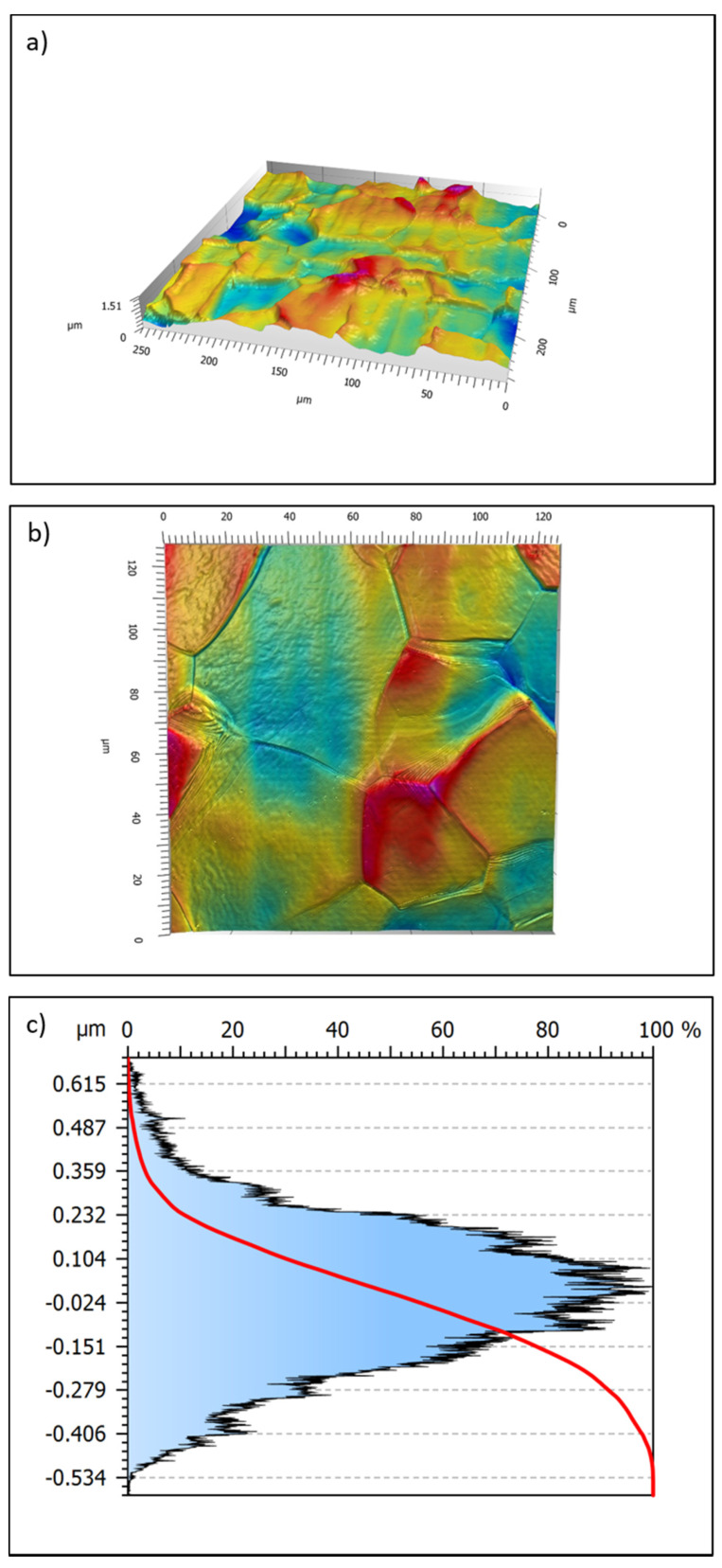
Confocal microscope images of EB10 sample; 50× (**a**) and 100× (**b**) magnifications. In the frame (**c**) the Abbott–Firestone curve is reported.

Confocal microscopy images of the EB10 sample are reported in Figure 3. Grooves almost 10 µm large, separated by slightly protruding ridges, are observable. Grain growth bands at the grain boundaries, formed during solidification, are well visible. Fast solidification and travelling of the electron beam generate a strong gradient of temperature [47], affecting the growth of the crystal. The thermal gradient, interface energies and surface tensions, generated in vacuum, result in an irregular topography, especially at the vicinity of some grain boundaries where an elevation of the grain surface is noticeable. From the topographical standpoint, the e-beam treated surface (EB10) shows the following parameters: Sa = 0.157 µm; Sq = 0.197 μm; Ssk = 0.04; Sku = 2.99. The Sa value is in line with the Ra value obtained through a contact profilometer reported and discussed below. It is here interesting to underline that the topographical profile is almost gaussian. In fact, the ratio between the Sq and Sa values is close to 1.25, while the Ssk and Sku values are close to 0 and 3, respectively. All these parameters describe a smooth surface without sharp spikes or deep and narrow valleys. This aspect is of relevance to favor fibroblast adhesion, as discussed below. The Abbott–Firestone curve of EB10 is reported in Figure 3c. This curve is a representation of the probability Amplitude Density Function—it reports which is the probability to find a point on the surface at the reported different heights and evidences the position of the average line. It confirms an almost symmetrical ratio between positive and negative features on the surface, with a small prevalence of valleys about 0.3–0.4 µm deep, as expected because of the presence of the grooves.

Nevertheless, this investigation technique is not able to make clear the surface nano-topography observed in Figure 2.

**Figure 4:**
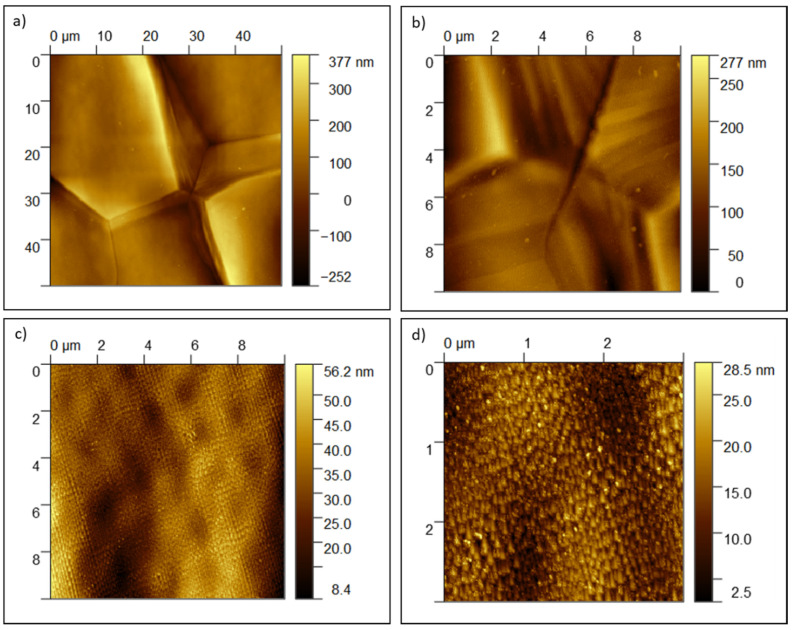
AFM images of Ti15Mo-EB10 surface: (**a**) topography of different grains with growth bands at grain boundaries (50 × 50 µm scan); (**b**) higher magnification of the growth bands in Figure 4a (10 × 10 µm scan); (**c**) nanosteps in the center of a grain ((10 × 10 µm scan); (**d**) detail of the nanosteps’ texture (3 × 3 µm scan).

To better characterize the treated surfaces from the topographical standpoint, AFM was used. The obtained AFM images are reported in Figure 4. Due to the high magnification, grooves are slightly visible only on the image reported in Figure 4a. On the other hand, the grain growth bands are well visible in Figure 4a,b. The mean surface roughness in the proximity of these waves is at about 27 nm. Moreover, nano-steps are evident in Figure 4c. This nano-texture is present on many grains and covers all their surface, but it is absent on some grains, and it shows different orientations on neighbor grains, revealing a correlation with the crystallographic orientation of the grain. The mean surface roughness in the proximity of these nano-steps is at about 7 nm. Finally, in some cases, these nano-steps highlight also a smaller and more complex texture (Figure 4d).

The same images acquired in Kelvin Probe mode do not evidence any difference in the surface potential in correspondence of the waves at grain boundaries or the nano-steps. This indicates that the observed effect is of topographical and crystallographic nature, but not correlated to any chemical difference.

### 3.2. Crystalline Structure Evaluation

**Figure 5:**
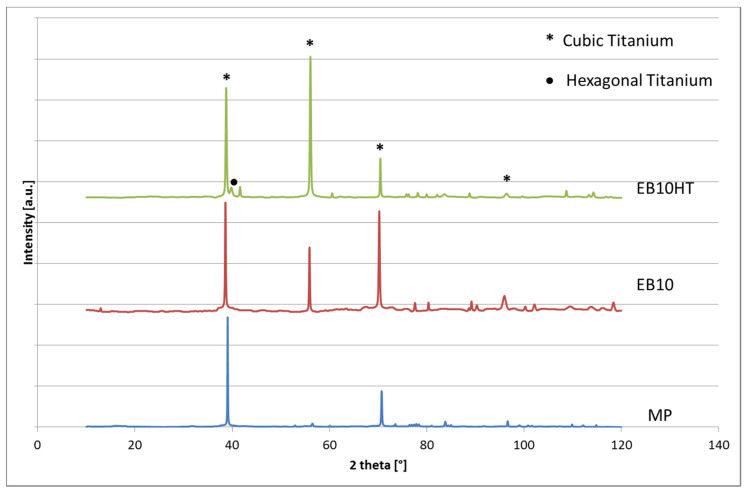
XRD patterns.

XRD patterns of the e-beam (EB10, EB30) and heat-treated (EB10HT) surfaces are reported in Figure 5; the pattern of the polished surface is reported as a reference (MP). In accordance with the expectations for a metastable beta titanium alloy, all the patterns evidence the characteristic peaks of cubic β titanium (MP and EB10 samples). A peak of hexagonal α titanium appears after the thermal treatment (EB10HT), in agreement with the precipitation of α phase.

### 3.3. Surface Roughness

The mean roughness (Ra) of the mechanically polished (MP), e-beam (EB10, EB30) and heat-treated (EB10HT) surfaces is reported in Table 1. As expected, surface texturing increases roughness with respect to the mirror polished samples because of the introduction of the grooves. However, as previously reported for Ti-cp and Ti6Al4V, the final roughness value of the treated samples is lower (or slightly higher, in the case of EB10HT) than the threshold reported in the literature to avoid an increase in bacterial adhesion (0.2 µm) [48,49,50]. The Ra values here obtained on Ti15Mo samples are close to the ones obtained on Ti-cp and Ti6Al4V [32,33]. An increment of Ra after the heat treatment agrees with morphology of the surface reported in Figure 1, where grain boundaries and grains on different levels were much more evident than on MP and EB10.

### 3.4. Surface Wettability

Contact angle results are reported in Table 1 for the same samples. A mirror polished Ti15Mo alloy appears slightly more hydrophobic compared to Ti-cp and Ti6Al4V [31,33]. The effect of EB texturing, however, seems the same than the one observed on the other substrates: a slight increase in the contact angle, attributable to the increase in the surface roughness compared to the mirror polished samples.

### 3.5. Biological Characterizations

#### 3.5.1. Cells’ Contact Guidance

**Figure 6:**
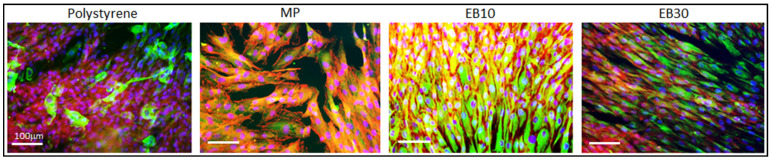
Cells’ morphology and orientation after 48 h cultivation. A random orientation was observed for the polystyrene and MP specimens, while in EB10, cytoskeletons were oriented following surface grooves as stained by F-actin (red, phalloidin) and intermediate filaments (green, vimentin). The number of nuclei (stained in blue by DAPI) suggested for a comparable colonization.

Cells’ morphology and orientation after 48 h of cultivation onto specimens’ surface are shown in Figure 6. Cells appeared as randomly oriented both on the polystyrene control and on mirror polished (MP) surfaces as revealed by the phalloidin staining (red) to indicate cytoskeleton F-actins filaments as well by the vimentin (green) representative for the intermediate filaments. On the other hand, a complete alignment can be observed on EB10 samples, while only partial orientation can be observed on EB30 samples. The results are in accordance with those obtained on Ti-cp and Ti6Al4V [32,33], confirming specimens EB10 (10 µm grooves) as the optimal width for gingival fibroblast alignment.

#### 3.5.2. Antifouling Properties

**Figure 7:**
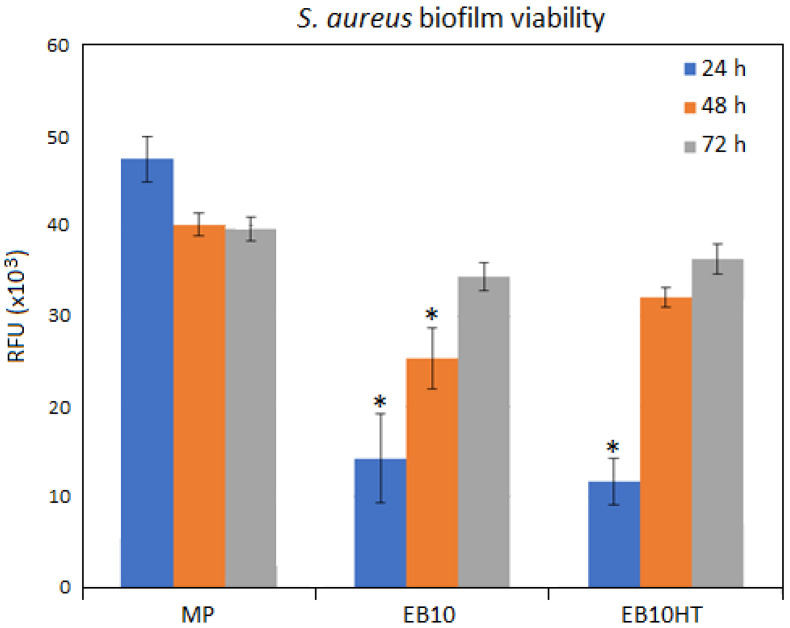
Bacterial adhesion evaluated in terms of metabolic activity. The grooved specimens (EB10 and EB10HT) resulted as significantly less contaminated after 24 h in comparison with mirror polished (MP) control (*p* < 0.05, indicated by *). Bars represent means and standard deviations. Replicates = 3.

Bacterial adhesion results are reported in Figure 7. It can be observed that e-beam texturing does not increase bacterial attachment, and e-beam texturing and thermal treatment reduces bacterial adhesion, especially at short contact times (24 h, *p* < 0.05 indicated by *) in accordance with an antifouling mechanism more than with an anti-bacterial one, as expected for surfaces that have no active antibacterial agent.

## 4. Discussion

E- beam structuring was successfully applied to a beta Ti15Mo alloy to obtain parallel grooves with 10 or 30 µm spacing and surface nano-structuring.

As previously observed by the authors on Ti-cp and Ti6Al4V alloys, [32,33], 10 µm spaced grooves (EB-10 samples) are the most effective in cell guidance (Figure 6). This phenomenon can be associated with cell dimension (15–25 µm for gingival fibroblasts, here applied as representative for the soft tissue being in contact with the collar of a dental implant), which is comparable with the groove width. In this case, fibroblasts should be in contact with the walls and the bottom of the groove. Other cell populations, such as osteoblasts, were not tested because they are not expected to colonize the collar of the implant, whereas undifferentiated mesenchymal stem cells migrating to the injured site hold a fibroblast-like morphology, behaving as fibroblasts.

Narrower grooves (few microns) can induce higher adhesion and alignment rates on fibroblasts, but a fibrotic response can come from the longer times of activation state of the cells. The here obtained grooves can effectively and positively affect cell adhesion and orientation by means of a contact guidance phenomenon [51,52,53]. The possibility to control the cells’ orientation can be of particular interest for dental implants; in fact, it has been previously demonstrated that oriented cell bundles can assure a more stable gum sealing than the random ones [28]. An effective sealing strongly curtails the risk of periodontal pocket formation, which is the cause of epithelial downgrowth and bacterial infection. Moreover, fibroblasts are known to be the first promoters of the inflammatory cascade through the secretion of chemokines IL6, IL8 and monocyte chemoattractant protein 1 (MCP1) [34]. Contact guidance represents a suitable tool to ease implant colonization, thus preventing a pro-inflammatory signaling cascade as it occurs in case of fibroblasts’ poor surface adaptability.

To be used in medical devices (e.g., implant collars), grooves should not increase bacterial adhesion compared to the gold standard mirror polished surface. Bacterial adhesion tests (Figure 7) evidenced that *S. aureus* adhesion is even reduced compared to mirror polished surfaces. This result indicates that, as for Ti-cp and Ti6Al4V [32,33], e-beam structuring introduces an antiadhesive effect for bacteria on Ti15Mo surfaces. Even if the effect of microfouling prevention was already observed by the authors on different titanium-based materials, this investigation looks to clarify the case in a beta titanium alloy.

Looking at the SEM observation, it seems that the formation of nano-stairs can be one of the key points for the reduction of bacterial adhesion. Bacteria are smaller in size than fibroblasts (1–2 µm) and present a less deformable membrane compared to eukaryotic cells. Consequently, their adhesion can be more difficult on nano-textured surfaces [39,44,54]. On smooth surfaces (Ra lower than 0.2 µm), bacteria are exposed to the shear forces that can cause their removal; however, bacterial flagella have the ability of perceiving nano roughness and shape differences [51]. As previously demonstrated, the presence of these surface nanotextures represents a strong limitation for bacteria adhesion as they strongly restrict the availability of berth points; therefore, this limited free-area impairs the adhesion of high-density bacteria [43,44]. Moreover, experiments were conducted by using the Gram-positive (Gram+) *S. aureus* as pathogen reference, thus likely enhancing the effect of the nano-topography. In fact, Gram-positive bacteria hold a rigid peptidoglycan layer that does not allow fluidic movements. Accordingly, they are not able to adapt to the nanotextured topography, thus failing surface adhesion.

On the opposite side, fibroblast adhesion was not affected by the presence of the nano-stairs/step and their spread was mostly influenced by the micro-topographies represented by the oriented grooves. These findings are in line with previous literature showing that fibroblast adhesion and spread can be influenced by surface micro-topographies, whereas the introduction of secondary nano-structures (over the micro-ones) did not induce a further modification of cells’ circularity, elongation and perimeter [44,45]. Thus, the observed antifouling effect was limited to the bacteria.

Other factors that can affect bacterial attachment beyond nano-topography, such as the surface residual stress and modification of surface composition (element partitioning due to selective evaporation), cannot be excluded, although they would not have a significant role. A similar anti-fouling effect has been observed on Ti-cp [32], where no modification of surface composition is expected because of evaporation. Surface stress could have a role both in the anti-fouling and in the contact guidance effect, affecting reorganization of the extra cellular matrix (ECM) into structures which, in turn, affect the behavior of the individual cells [54]. Micro or nano-topography can also induce changes in surface energy; a larger hydrophobicity could be expected on regularly micro-structured surfaces and even more on surface features at the nanoscale, but this is not the case as shown by the contact angle data. In any case, it is already reported in the literature that even if both surface energy and topography of a surface affect bacterial adhesion on it, a predominant role is played by topography [54]. Surface wettability analysis reported high values of contact angles (80–90°); in fact, it is well-known that angles >60° do not represent an optimal condition for biomedical implants repopulation. However, previous studies reported contact angles similar to those here obtained, indicating that the main effect towards fibroblasts is a slowdown of proliferation over time, without inhibition of adhesion and growth [45,55].

The remarkable result of this research is to demonstrate that the peculiar nano-topography due to e-beam surface structuring is related to the crystallographic orientation of the grains, which is obtainable both on an alpha (such as c.p. Ti) [32], alpha-beta (such as Ti6Al4V) [33] and beta (such as Ti15Mo) alloys; this is also confirmed by the unchanged behavior of EB10HT with respect to EB10 even if the alpha phase is formed during the heat treatment.

## 5. Conclusions

Electron beam technology has been successfully applied to Ti15Mo alloys for the obtainment of parallel grooves with 10 or 30 µm spacing. We found that 10 µm spaced grooves are able to support gingival fibroblast adhesion and alignment without increasing bacterial adhesion. Moreover, e-beam structuring induces anti-adhesive properties for *S. aureus* bacteria. The peculiar nanotexture developed at the surface after EB treatment seems to have a major role in this behavior. However, the mechanism beyond this action is worthy of further investigations.

The possibility to simultaneously obtain such micro-scaled grooves and nano-structures by means of e-beam technology represents an effective strategy to favor cell adhesion and to reduce bacterial contamination without the use of active agents (often related with cytotoxicity concerns and regulatory complications) or in a synergic way with a low amount.

## Figures and Tables

**Table 1:** Roughness and wettability.

	Ra (µm)	Contact Angle (°)
MP	0.027 ± 0.003	82 ± 2
EB10	0.179 ± 0.033	82 ± 6
EB30	0.243 ± 0.009	95 ± 5
EB10HT	0.350 ± 0.070	93 ± 3

## Data Availability

Data sharing not applicable.
